# Symptoms of titanium and nickel allergic sensitization in orthodontic treatment

**DOI:** 10.1186/s40510-020-00318-4

**Published:** 2020-07-01

**Authors:** Martina Zigante, Marijana Rincic Mlinaric, Marija Kastelan, Vjera Perkovic, Magda Trinajstic Zrinski, Stjepan Spalj

**Affiliations:** 1 Department of Orthodontics, University of Rijeka, Faculty of Dental Medicine, Kresimirova 40, 51000 Rijeka, Croatia; 2 Orthodontic Office Rincic Mlinaric, Katarine Zrinske 1, 23000 Zadar, Croatia; 3 Department of Dermatovenereology, University of Rijeka, Faculty of Medicine, Brace Branchetta 20, 51000 Rijeka, Croatia; 4 Department of Dental Medicine 1, J.J. Strossmayer University of Osijek, Faculty of Dental Medicine and Health, Crkvena 21, 31000 Osijek, Croatia

**Keywords:** Allergic sensitization, Hypersensitivity, Titanium, Nickel, Orthodontics

## Abstract

**Aim:**

The study aimed to evaluate to which extent self-reported symptomatology, age, and sex are predictors of titanium and nickel allergic sensitization in patients in treatment with fixed orthodontic appliances.

**Methods:**

The study analyzed 228 subjects aged 11–45 years (median 18, interquartile range 16–22); 68% of them were females, and 52% were adolescents. The allergic sensitization testing included epicutaneous patch test to titanium, titanium dioxide, titanium oxalate, titanium nitride, and nickel sulfate. The questionnaire on symptoms potentially linked to titanium and nickel sensitization was used.

**Results:**

Prevalence of the allergic sensitization to titanium in patients undergoing orthodontic treatment was 4% (2% only to titanium without nickel) while to nickel 14% (12% nickel without titanium). Hypersensitivity to both metals at the same time was present in 2% of subjects. Sensitization to nickel was more common in females than in males (17 vs. 8%) and much more common in adults than in adolescents with small effect size (20 vs. 8%; *p* = 0.013). Sensitization to titanium was more common in females than in males (6 vs. 1%) with no difference in age. Multiple logistic regression analysis revealed that adult age increases the odds for being sensitized to nickel for 2.4 × (95% CI 1.1–5.6; *p* = 0.044) while watery eyes for 3.7 × (95% CI 1.2–11.1; *p* = 0.022). None of the symptoms were significant predictors of titanium sensitization.

**Conclusion:**

Allergic sensitization to titanium and nickel are not very frequent in orthodontic patients, and self-reported symptomatology is a weak predictor of those sensitizations.

## Introduction

Orthodontic treatment mostly implies fixed orthodontic appliance which consists of metal brackets bonded to tooth surface and archwire connecting them. Average treatment duration is between 2 and 3 years; therefore, safety and interactions of orthodontic materials with saliva, food, oral hygiene products, and oral and dental tissues should be kept in mind. Nickel- and titanium-containing alloys are often used in orthodontics. Nickel allotment in orthodontic alloys varies from 8% in stainless steel to 50% in nickel-titanium (NiTi) alloy [1]. Titanium is also present in orthodontic appliances from 50% in NiTi alloys up to 80% in titanium-molybdenum alloy and 90% titanium-aluminum-vanadium alloy used for orthodontic mini implants.

Hypersensitivity is an excessive, inadequate reaction of the immune system to an otherwise harmless antigen [2]. Hypersensitivity reactions lead to tissue damage and can cause serious conditions and illnesses. Usually, they do not lead to symptomatic reactions at the first exposure, but upon re-exposure to the causative antigen [3]. The most common form of allergic reactions to dental biomaterials in the oral cavity is contact allergic reaction, late type of hypersensitivity reaction, or cell-mediated (type IV) hypersensitivity [4, 5]. Positive epicutaneous test signifies allergic sensitization while diagnosis of allergy can be established only when clinical signs are present and when removing of the allergen causes regression of symptoms.

Nickel is a metal widespread in the environment, an essential nutrient, but it is also a very common contact allergen [6]. Nickel allergy occurs more frequently than allergies to other metals altogether. The prevalence of nickel allergy has been estimated up to 28.5% of the general population and cannot be considered as low potential risk [7, 8].

Due to its high biocompatibility, titanium was previously considered to have no allergic potential; however, some authors report that titanium can be erosive if it co-exists with other types of metal or when it is exposed to fluoride ions in the acidic environment [9–11]. This suggests that during orthodontic treatment which includes presence of several types of metal alloys in the oral cavity media, such reactions may occur. There is also an increasing number of cases in which titanium allergy is reported in patients with implanted cardiac pacemaker, joint endoprosthesis, endovascular stents, surgical staples, and dental implants [12–16]. However, allergic sensitization to titanium alloys has not been systematically studied.

The aims of this study were to assess the prevalence of the allergic sensitization to titanium and nickel in patients undergoing orthodontic treatment with fixed appliances containing titanium and nickel and to evaluate to which extent self-reported symptomatology, age, and sex are related to them. Hypotheses of this study were that both titanium and nickel allergic sensitizations are more frequent in females and in adults and that a self-reported symptom of oral burning is the most powerful predictor of both sensitizations.

## Materials and methods

A total of 250 subjects undergoing orthodontic treatment in three orthodontic offices in Croatia were asked to participate in this observational cross-sectional study. Assuming the prevalence of allergies of 15–20% (95% confidence intervals 10–25%) and precision of 5% estimated, sample size was calculated to be 196–246, so it was decided to recruit 250 participants. Inclusion criteria were treatment with fixed orthodontic appliance, while exclusion criteria were diabetes, endocrine and autoimmune diseases, and practicing water sports. All patients were treated with the same kind of metallic brackets (Ortho Classic, USA) and archwires (GAC International, Japan). Orthodontic forces were mild and continuous, part of the patients was in the phase of treatment involving intermaxillary elastics and in case of the space closure sliding mechanics was used. From a total of 250 subjects invited to participate, 22 (9%) dropped out because of the practicing of water sports or uncomplete questionnaires on symptoms potentially linked to titanium and nickel allergies. Therefore, 228 participants were included in the study in the age of 11 to 45 years (median 18, interquartile range 16–22); 68% of them were females, and 52% were adolescents. None of the subjects with incomplete questionnaire on oral changes and symptoms potentially linked to titanium and nickel allergies were allergic to titanium nor to nickel.

Epicutaneous testing and administration of questionnaires were performed in the period from 2 months after bonding the appliance up to 24 months of treatment. In order to control and address potential sources of selection and recall bias, the same questionnaire form was applied and potential ambiguities were addressed the same way; it was explained that the testing is nothing scary or harmful; we asked participants not to overemphasize the self-reported symptoms, and we tried to keep track of the duration of orthodontic treatment, so the participants could remember if they experienced different symptoms since they had been treated with fixed orthodontic appliances in comparison with before the treatment. All participants were tested for allergic sensitization, and testing included the application of an epicutaneous patch test to nickel sulfate, titanium, titanium dioxide, titanium oxalate, and titanium nitride with petrolatum used as control (Chemotechnique Diagnostics, Vellinge, Sweden). The skin was cleaned by wiping with cotton wool soaked in medical petrol (Medimon, Split, Croatia) in order to degrease it. The allergens were applied on the upper arm skin and left under occlusion for 2 days, and the patients were instructed not to wet the area. Evaluations of the skin reactions were performed three times—on the second, fourth, and seventh day after applying the patches, as suggested by the manufacturer. Skin reactions were evaluated according to Reading Plate (Chemotechnique Diagnostics, Vellinge, Sweden).

The questionnaire on oral changes and symptoms potentially linked to titanium and nickel allergies was used. Closed type questions with dichotomous answers were applied, meaning that subject could choose between yes and no answer (0 = no; 1 = yes), whereas all questions were related to the period since the patient started the orthodontic treatment. Questionnaire for patient-reported symptoms related to the period of orthodontic treatment included questions on possible onset of the following symptoms: oral burning feeling, changed sense of taste, weak sense of taste, metal taste, lack of saliva, weak sense of smell, changed sense of smell, swollen tongue feeling, skin changes, swelling in the oral cavity or facial swelling, abdominal pain, diarrhea, bloating, frequent headaches, tinnitus, dizziness, nose leaking, watery eyes, and frequent sneezing. In case oral burning was a reported symptom, its intensity was evaluated by the visual analog scale where 0 = no feeling of oral burning and 100 = the strongest possible feeling of oral burning. Patients were determining the intensity of burning by placing a mark on a visual analog scale.

Prevalence of allergic sensitization was estimated with 95% confidence intervals (CI) [17]. Predictors of allergies were explored by applying Fisher’s exact test and logistic regression, and odds ratios (OR) with 95% CI were calculated. Effect size for Fisher’s test was quantified by means of Cramer V. For interpretation, the Cohen criteria were used 0.1–0.3 = small, 0.3–0.5 = medium, 0.5–0.7 = large, and > 0.7 very large effect size [18]. In the interpretation, OR = 1.5 was considered mild, while moderate > 3, and large > 9 [19]. All statistical analyses were performed in the statistical software IBM SPSS 22 (IBM Corp, Armonk, USA).

## Results

Prevalence of the allergic sensitization to titanium and/or nickel in patients undergoing orthodontic treatment was 16% (95% CI 12–22%), less often to titanium than nickel (4%; 95% CI 2–8% vs 14%; 95% CI 10–19%). Isolated sensitization on titanium (without nickel) was detected in 2% of subjects (95% CI 1–5%) while isolated sensitization on nickel (without titanium compounds) was detected in 12% of subjects (95% CI 8–17%). Hypersensitivity to both metals at the same time was present in 2% of subjects (95% CI 1–5%). Females were more often sensitized to nickel than males (17 vs 8%) and much more adults than adolescents with small effect size (20 vs 8%; *p* = 0.013; *V* = 0.169; OR = 2.8; 95% CI 1.2–6.1). A titanium sensitization was also more common in females than in males (6 vs. 1%) but equal in adolescents and adults.

In univariate analyzes, allergic sensitization to titanium and/or nickel was related to female sex, adult age, self-reported weak sense of taste, self-reported weak sense of smell, swelling of the tongue and/or face, and watery eyes (Table 1).

**Table 1:** Univariate predictors of nickel and/or titanium sensitization

Variable	Category		Sensitization		p*	V**	OR*** (95% CI)
			Absent	Present			
Sex	Male (0)	*N*	66	6	0.033	0.145	2.7 (1.1–6.9)
		%	91.7%	8.3%			
	Female (1)	*N*	125	31			
		%	80.1%	19.9%			
Age	Adolescents (0)	*N*	106	13	0.030	0.150	2.3 (1.1–4.8)
		%	89.1%	10.9%			
	Adults (1)	*N*	85	24			
		%	78.0%	22.0%			
Weakened sense of taste	No (0)	*N*	188	34	0.023	0.151	5.5 (1.1–28.6)
		%	84.7%	15.3%			
	Yes (1)	*N*	3	3			
		%	50.0%	50.0%			
Weakened sense of smell	No (0)	*N*	189	34	0.031	0.178	8.3 (1.3–51.8)
		%	84.8%	15.2%			
	Yes (1)	*N*	2	3			
		%	40.0%	60.0%			
Swelling of tongue and/or face	No (0)	*N*	167	27	0.040	0.150	2.6 (1.1–6.0)
		%	86.1%	13.9%			
	Yes (1)	*N*	24	10			
		%	70.6%	29.4%			
Watery eyes	No (0)	*N*	175	29	0.034	0.159	3.0 (1.2–7.7)
		%	85.8%	14.2%			
	Yes (1)	*N*	16	8			
		%	66.7%	33.3%			

*Fisher’s exact test

**Effect size

***Odds ratio

All predictors had small effect sizes; self-reported weak sense of taste and smell and watery eyes were moderate odds, while the rest of the predictors were mild odds. However, putting those predictors altogether in the logistic regression model in order to control their interrelationships, only sex and watery eyes remained significant. The odds are 2.7 × higher that females will be allergically sensitized compared to males (95% CI 1.1–6.9; *p* = 0.043), while subjects with watery eyes have 3.1 × higher odds of being sensitized (95% CI 1.1–8.3; *p* = 0.028).

When analyzed separately, only adult age and watery eyes were related to the allergic sensitization to nickel in univariate analyzes (Table 2).

**Table 2:** Univariate predictors of nickel sensitization

Variable	Category		Sensitization		p*	V**	OR*** (95% CI)
			Absent	Present			
Watery eyes	No	*N*	179	25	0.033	0.149	3.0 (1.1–7.8)
		%	87.7%	12.3%			
	Yes	*N*	17	7			
		%	70.8%	29.2%			
Age	Adolescent	*N*	109	10	0.013	0.169	2.8 (1.2–6.1)
		%	91.6%	8.4%			
	Adult	*N*	87	22			
		%	79.8%	20.2%			

*Fisher’s exact test.

**Effect size

***Odds ratio

When both predictors were put together in a logistic regression model, adult age decreased its odd for sensitization to nickel from 2.8 to 2.4 (95% CI 1.1–5.6; *p* = 0.044), while self-reported watery eyes became an even more stronger predictor, increasing the odds from 3.0 to 3.7 (95% CI 1.2–11.1; *p* = 0.022).

Predictors of sensitization to titanium in univariate analyses were self-reported sense of oral burning, change of sense of taste, weakened sense of taste, weakened sense of smell, swelling of the tongue and/or face, and vertigo (Table 3).

**Table 3:** Univariate predictors of titanium sensitization

Variable	Category		Sensitization		p*	V**	OR*** (95% CI)
			Absent	Present			
Oral burning	No (0)	*N*	209	8	0.022	0.152	5.8 (1.1–31.4)
		%	96.3%	3.7%			
	Yes (1)	*N*	9	2			
		%	81.8%	18.2%			
Changed sense of taste	No (0)	*N*	203	7	0.035	0.176	5.8 (1.4–24.7)
		%	96.7%	3.3%			
	Yes (1)	*N*	15	3			
		%	83.3%	16.7%			
Weakened sense of taste	No (0)	*N*	214	8	0.024	0.232	13.4 (2.1–84.1)
		%	96.4%	3.6%			
	Yes (1)	*N*	4	2			
		%	66.7%	33.3%			
Weakened sense of smell	No (0)	*N*	215	8	0.016	0.260	17.9 (2.6–122.6)
		%	96.4%	3.6%			
	Yes (1)	*N*	3	2			
		%	60.0%	40.0%			
Swelling of tongue and/or face	No (0)	*N*	189	5	0.008	0.211	6.5 (1.8–23.9)
		%	97.4%	2.6%			
	Yes (1)	*N*	29	5			
		%	85.3%	14.7%			
Vertigo	No (0)	*N*	210	7	0.008	0.252	11.3 (2.5–51.7)
		%	96.8%	3.2%			
	Yes (1)	*N*	8	3			
		%	72.7%	27.3%			

*Fisher’s exact test.

**Effect size

***Odds ratio

However, in multiple logistic regression model, all predictors became insignificant.

Sneezing, rhinorrhea, headache, bloating, digestion problems and diarrhea, abdominal pain, dry mouth, metallic taste in the mouth, changes of the sense of smell, tinnitus, or skin changes were not related to the allergic sensitization to nickel, sensitization to titanium, nor to both of those metals.

## Discussion

Based on the results of this study, allergic sensitizations to titanium and nickel in patients undergoing orthodontic treatment are not very frequent, with titanium sensitization less prevalent than nickel, and there are few typical symptoms accompanying them. Titanium sensitization is of special interest in orthodontics, since titanium brackets are considered as an alternative in patients with nickel hypersensitivity, which might not be safe considering that some patients with hypersensitivity on nickel are also sensitized on titanium. In case of patient medical history of nickel hypersensitivity, ceramic brackets might be safer alternative.

Until nowadays, considerations regarding oral dosage of metals or allergens generally leading to oral contact hypersensitivity outburst are not clarified. Considering the interval of treatment in which epicutaneous testing on titanium and nickel was performed, some patients were in the phase of treatment involving intermaxillary elastics. Intermaxillary elastics and application of higher forces can cause more cracking of the protective corrosion layer on the surface of the archwire leading to more pronounced discharge of its components in the oral cavity. However, in sensitized patients, such usage of intermaxillary elastics and application of higher forces could trigger allergic contact reaction outburst due to higher metal release.

Symptoms related to nickel or titanium allergic sensitization in univariate analyses of present research are a weak sense of smell and/or taste, swellings of the tongue or face, and watery eyes. Previous data also suggest that gustatory impairment, sensory dysfunctions, swelling of the lip and face, and even tongue may be the symptoms of oral contact allergy [20–23]. The orofacial region has been associated with type I, III, and IV allergies. Metals can be bonded with native proteins to form haptenic antigens in their ionic form or can trigger degranulation of mast cells and basophils [24, 25]. Swelling of the tongue could be a manifestation of the type I allergy to metals, or it may be a subjective sensation of edema due to actual numbness of the tongue [26].

Nevertheless, when put together in multiple regression, our study showed that the symptom of watery eyes was the most important predictor of having allergic sensitization primarily related to nickel. So, by this statistical procedure, the effect of other variables was controlled for or was kept constant pointing to the most prominent symptom related to sensitization. Watery eyes were not described before in relation to nickel or titanium contact allergy. The allergic eye symptoms are mostly associated with atopy and type I hypersensitivity. Increase of lacrimation is among the symptoms of ocular allergic conjunctivitis. In the early phase, mast cells activate allergic inflammation, whereas in the late phase recruitment of inflammatory cells to the site of allergic inflammation occurs [27]. The exact pathological mechanism of how ingested nickel alone could provoke systemic symptoms remains unknown. It could be that sensitized subjects have immunological system more prone to other allergies as well so the symptomatology can be intertwined.

Several symptoms were related to allergic sensitization to titanium in univariate analyses, namely self-reported swelling of the tongue, change of sense of taste and smell, oral burning, and even vertigo. However, all predictors had small effect size and in multiple regression become insignificant, so they cannot be considered as reliable predictors of titanium allergic sensitization. One of the reasons is probably a small number of subjects with titanium allergic sensitization.

We did not confirm oral burning as a predictor of allergic sensitization, as expected, contrary to others [28]. It seems that oral burning is rather associated with nutritional deficiencies (iron, folic acid, vitamin B, or zinc), infection with *Candida albicans*, conditions such as diabetes mellitus or Sjögren syndrome or even psychological causes (e.g., depression, anxiety, somatoform disorders) more likely than with the allergy [29, 30]. Dry mouth can be an isolated symptom of oral contact allergy to nickel and other metals or a part of the oral burning syndrome and it can also appear in a more serious form of xerostomia [31, 32]. Our study did not show that dry mouth or oral burning could be the predictors of hypersensitivity probably due to its multifactorial background.

This study also did not relate sneezing, rhinorrhea, headache, bloating, digestion problems or diarrhea, abdominal pain, dry mouth, metallic taste in the mouth, changes in the sense of smell, tinnitus, or skin changes to nickel or titanium hypersensitivity. There are very few studies investigating these symptoms in the context of allergies to dental materials. Some authors reported sneezing as one of the respiratory manifestations of nickel allergy [33]. Rhinorrhea was not reported as a symptom of nickel nor of titanium allergy, but it was connected with an allergy to endodontic materials [34]. Literature suggests that metallic taste could be connected with nickel allergy [32, 35]. Tinnitus is reported as one of the possible symptoms of metal allergy, but to chromium [36].

Contrary to present research, some authors suggest that bloating, abdominal pain, digestion problems or diarrhea, and cutaneous changes can be systemic signs of nickel allergy associated with ingestion of nickel [31, 37]. Headaches are sometimes reported in subjects with oral contact allergy, namely to nickel, but they appear rarely and are not a specific sign of an allergy [31, 38].

Since this study was based on self-reported symptomatology, previous exposition to titanium and nickel from other sources (space maintainers, removable appliances, piercings, tattoos, etc.) was not taken into account. Sensitization in patients wearing other types of orthodontic appliances and mini implants should be investigated for titanium and nickel sensitizations. Also, immunohistochemical analyses of gingiva of sensitized patients and analyses of gingival crevicular fluid could shed new insights on these allergies.

## Conclusion

Allergic sensitization to titanium occurs more often than expected and therefore should not be overlooked. However, it is more uncommon than sensitization to nickel and there are no clear symptoms related to allergic sensitization to titanium and nickel. Titanium allergy needs to be further investigated.

## Data Availability

The datasets used and/or analyzed during the current study are available from the corresponding author on reasonable request.
